# Correction to: BIODICA: a computational environment for Independent Component Analysis of omics data

**DOI:** 10.1093/bioinformatics/btad597

**Published:** 2023-10-07

This is a correction to: Nicolas Captier, Jane Merlevede, Askhat Molkenov, Ainur Ashenova, Altynbek Zhubanchaliyev, Petr V Nazarov, Emmanuel Barillot, Ulykbek Kairov, Andrei Zinovyev, BIODICA: a computational environment for Independent Component Analysis of omics data, *Bioinformatics*, Volume 38, Issue 10, May 2022, Pages 2963–2964, https://doi.org/10.1093/bioinformatics/btac204

A change has been made to the author list.

